# Supplementary material for: Heterogeneity and transcriptome changes of human CD8+ T cells across nine decades of life
Source: Nat Commun. 2022 Sep 1;13:5128. doi: 10.1038/s41467-022-32869-x (PMC9436929; doi:10.1038/s41467-022-32869-x)
Supplement: Supplementary file 1 — Supplementary Information [file 41467_2022_32869_MOESM1_ESM.pdf]

Heterogeneity and transcriptome changes of human $CD8^+$ T cells across nine decades of life

Jian Lu[1*], Raheel Ahmad[1*], Thomas Nguyen[1], Jeffrey Cifello[1], Humza Hemani[1], Jiangyuan Li[1], Jinguo Chen[2], Siyi Li[1], Jing Wang[1], Achouak Achour[1], Joseph Chen[1], Meagan Colie[1], Ana Lustig[1], Christopher Dunn[1], Linda Zukley[3], Chee W. Chia[4], Irina Burd[5], Jun Zhu[6], Luigi Ferrucci[3], and Nan-ping Weng[1**]

[1] Laboratory of Molecular Biology and Immunology, National Institute on Aging, National Institutes of Health
[2] Center for Human Immunology, Autoimmunity and Inflammation (CHI), National Institutes of Health
[3] Translational Gerontology Branch, National Institute on Aging, National Institutes of Health
[4] Laboratory of Clinical Investigation, National Institute on Aging, National Institutes of Health
[5] Department of Obstetrics and Gynecology, Johns Hopkins University School of Medicine
[6] National Heart, Lung, and Blood Institute, National Institutes of Health

* These two authors contribute equally.

**Correspondence to Nan-ping Weng (Wengn@mail.nih.gov)

Supplementary materials:

Supplementary Figure 1 Analysis of human $CD8^+$ T cell subpopulations by scRNAseq and multi-color flow cytometry.
Supplementary Figure 2 $CD8^+$ T cell subpopulations in participants and their age changes.
Supplementary Figure 3 Gene Set Enrichment Analysis (GSEA) of differentially expressed genes in ten subpopulations of $CD8^+$ T cells
Supplementary Figure 4 Age-related increased and decreased genes in nine subpopulations of $CD8^+$ T cells
Supplementary Figure 5 Predicting cell age by two machine learning models
Supplementary Figure 6 Mutational analysis of $CD8^+$ T cell subpopulations and their change with age
Supplementary Figure 7 Overlap age-changed genes identified by MELR and MERF model

Supplementary Table 1 Information of participants' age, gender, number of CD8 T cells, composition of CD8 T cell subpopulations by scRNAseq and coverage by exome sequencing
Supplementary Table 2 Selected enriched gene expression function in CD8 T cell subpopulations
Supplementary Table 3 Antibodies used in this study
Supplementary Table 4 Sequences of primers used to construct antibody-derived tag (ADT) libraries

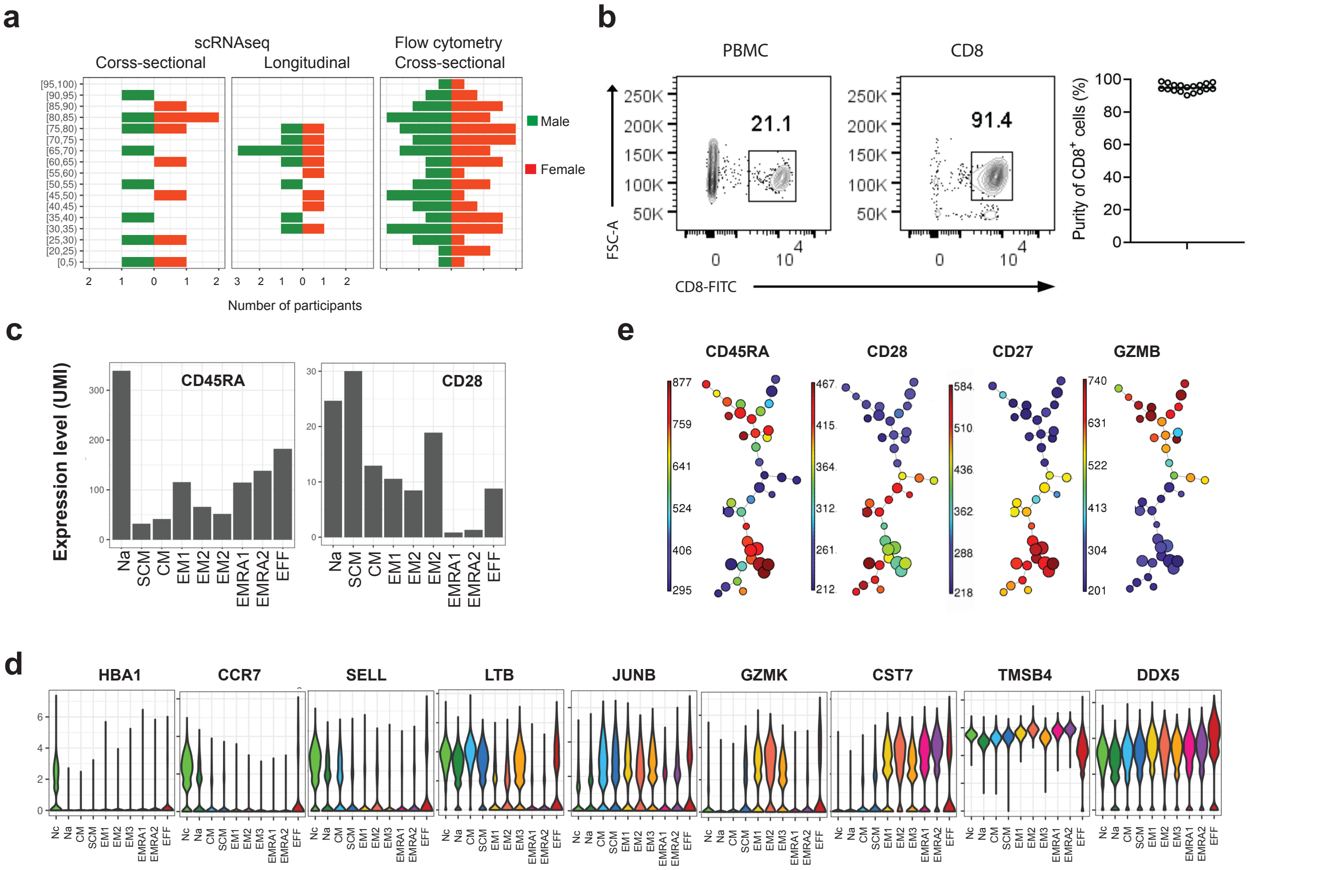

Supplementary Figure 1: Analysis of human CD8$^+$ T cell subpopulations by scRNAseq and multi-color flow cytometry. **a.** Distribution of Age and Sex of donors used in three cohorts used in study (Female – Orange, Male – Green). Donor age is binned for every 5 years. **b.** Left - FACS gating strategy for CD8$^+$ T cell sorting from a representative donor. Right – dot plot showing the distribution of CD8$^+$ T cell purity. **c**. Bar graphs showing the average expression of ADT libraries for CD45RA and CD28 antibodies in each cluster identified by scRNA-seq. **d.** DGEs of CD8$^+$ T cell subpopulations. The percentage of cells expressing each gene and average level of gene expression in each cluster shown**. e.** FlowSOM plots of selected markers from flow cytometry cohort.

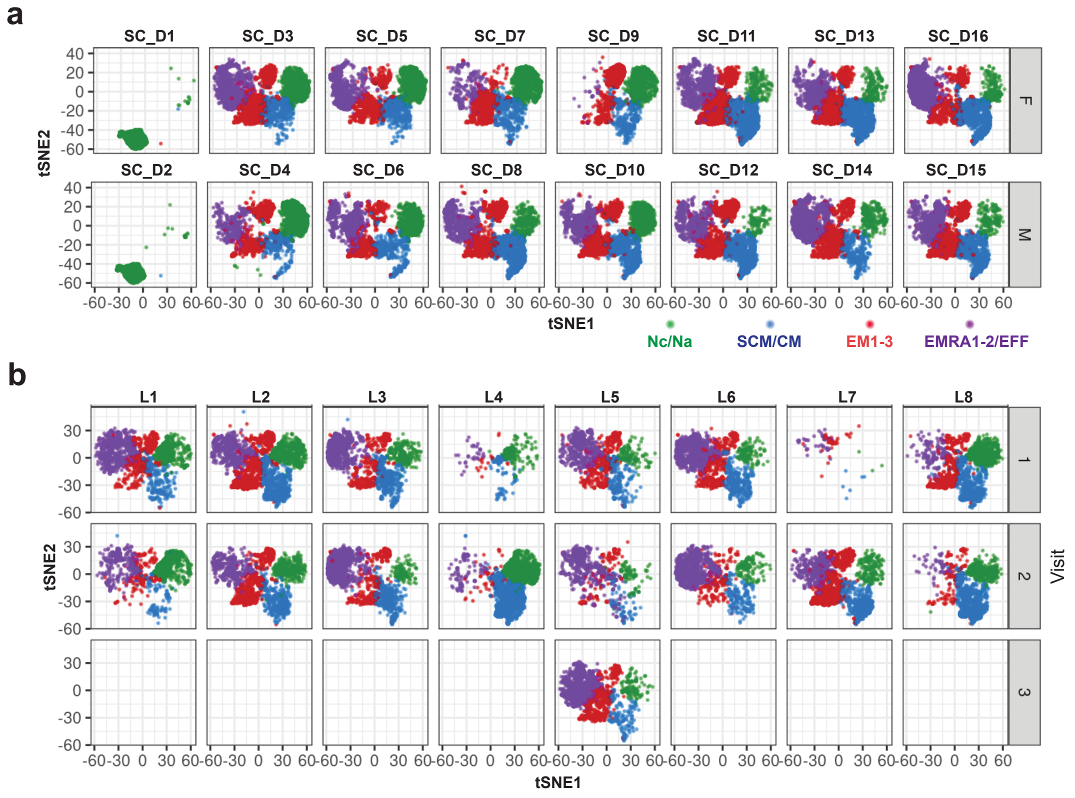

Supplementary Figure 2: CD8^{+} T cell subpopulations in participants and their age changes.
a. TSNE plots of single cells from each donor in the cross-sectional scRNA-seq cohort. Donors are ordered by age and stratified by sex (above – female; below - male). Plots are colored by major CD8^{+} T cell subset identities. **b**. TSNE plots of single cells from each donor in the longitudinal scRNA-seq cohort. Donors are ordered by age and stratified by visit number. Plots are colored by major CD8^{+} T cell subset identities.

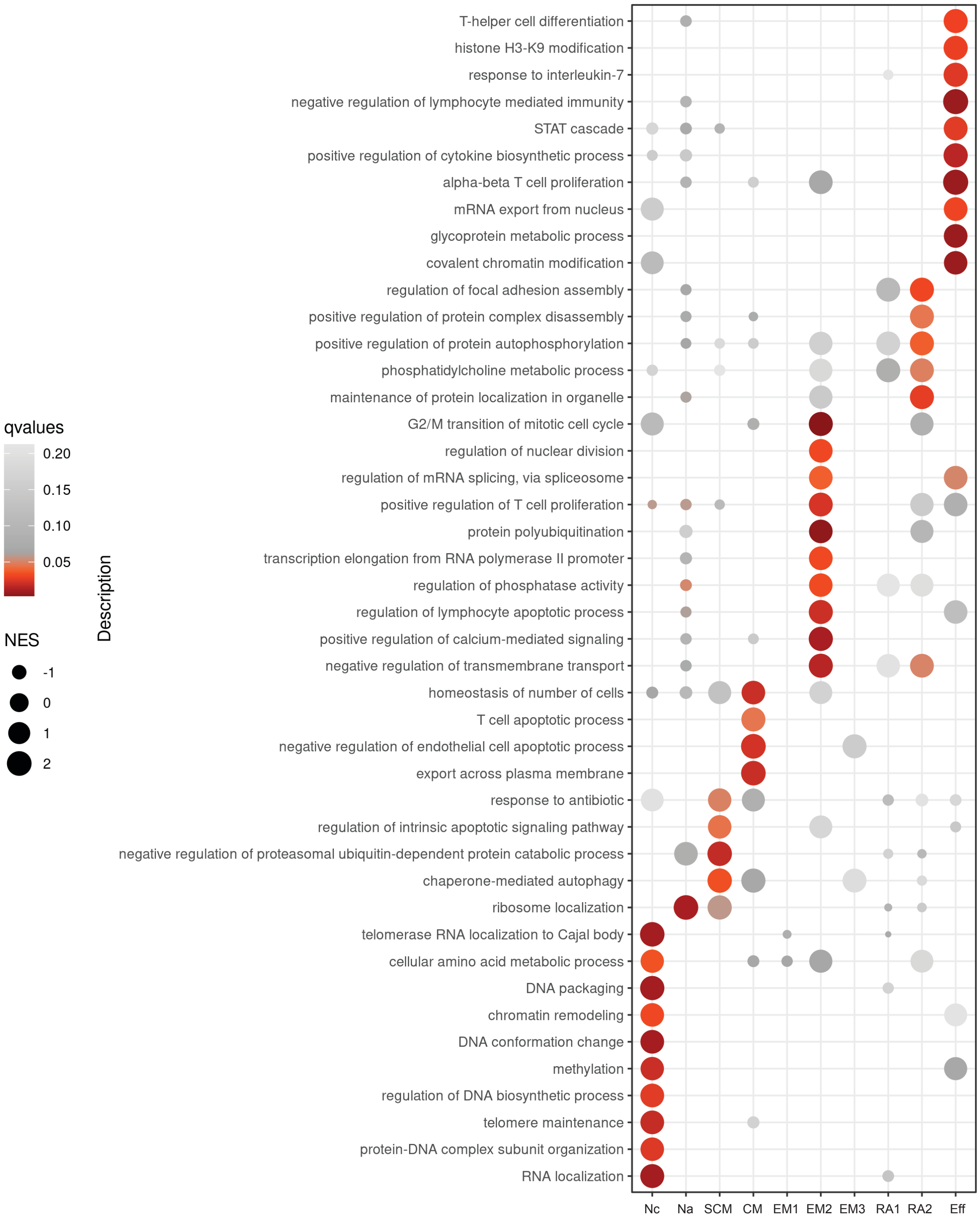

Supplementary Figure 3: Gene Set Enrichment Anlaysis (GSEA) of differentially expressed genes in ten subpopulations of CD8^{+} T cells. Bubble plot showing selected functional gene sets enriched in each cluster identified by scRNA-seq. Gene Set Enrichment Anlaysis (GSEA) was performed in the clusterprofiler R package with gene sets from the ‘Biological Processes’ groups. Size of bubbles represents normalized enrichment score (NES). The color of each bubble represents the significance of enrichment (qvalues).

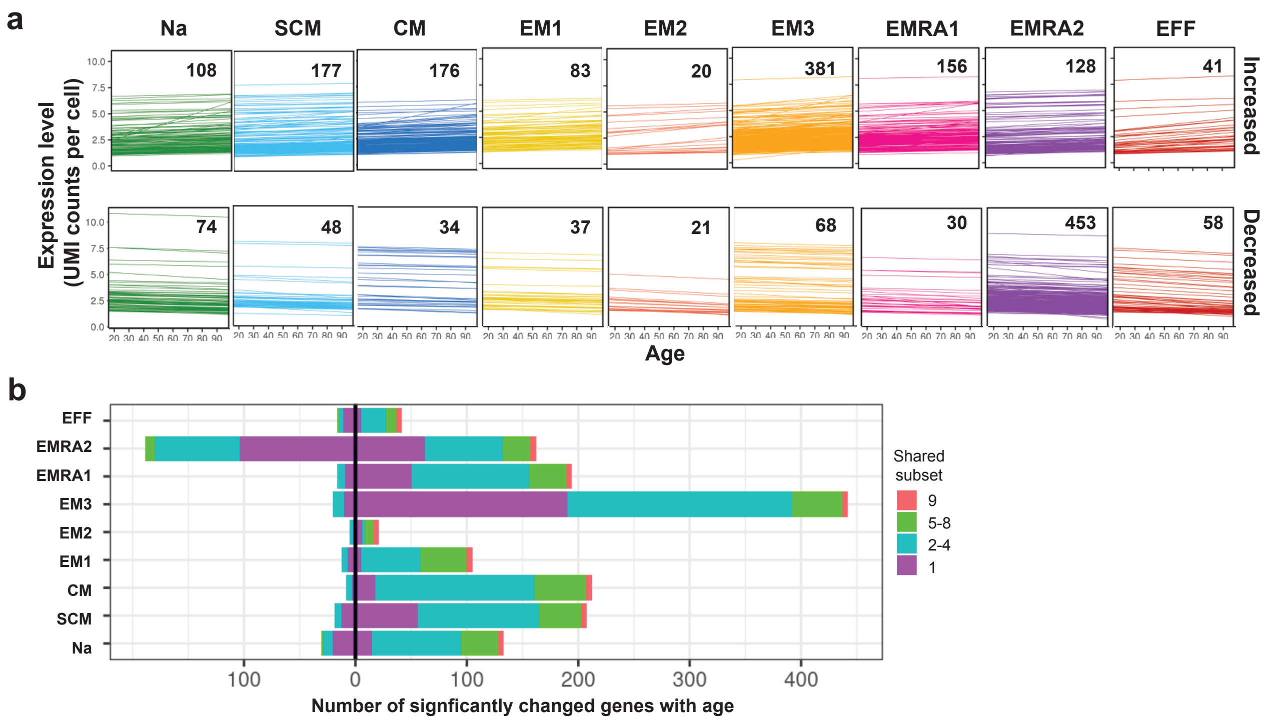

Supplementary Figure 4: Age-related increased and decreased genes in nine subpopulations of CD8$^+$ T cells. **a.** Line graphs showing the change in the average expression of significantly up-regulated age-related genes. Genes were identified based on a linear mixed effect regression model with a random intercept. Cells were grouped by donor in the longitudinal cohort; cells from the cross-sectional cohort were treated as one group. **b.** Line graphs showing the change in the average expression of significantly down-regulated age-related genes. Genes were identified based on a linear mixed effect regression model with a random intercept. Cells were grouped by donor in the longitudinal cohort; cells from the cross-sectional cohort were treated as one group. The number of significant genes is displayed in each graph. **c)** Bar graphs showing the shared and unique significant age-related gene expression changes in each cluster identified by scRNA-seq (left – downregulated; right – upregulated).

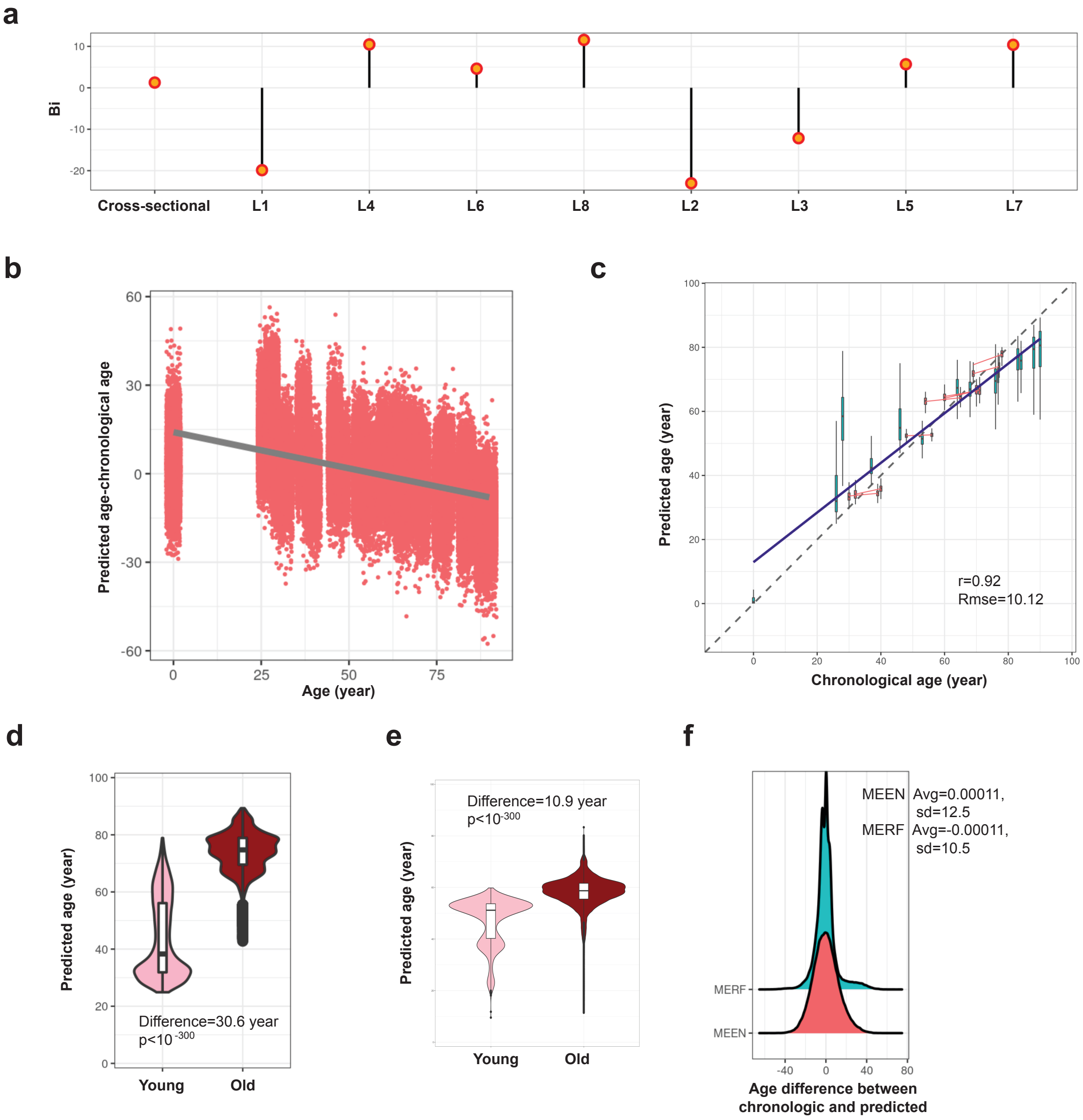

Supplementary Figure 5: Predicting cell age by two machine learning models. **a**. Lollipop plot showing the random effect coefficient for each longitudinal donor in the mixed effect elastic net model used to estimate single cell age. The random effect coefficient for cross-sectional donors was calculated as one group. **b**. Scatter plot showing the residuals from the mixed effect elastic net model ordered by chronological age. A least squares line was fit to identify the trend in residual value across age. **c.** Box plots showing the cross-validation predictions from the from the mixed effect random forest model ordered by chronological age. A least squares line was fitted to show the relationship between chronological age and predicted age of cells in the dataset. **d**. Violin plots showing predictions generated from the mixed effect random forest model on a test set of $CD8^+$ T cells from the validation dataset. Data are grouped into grouped into old (>70) and young (<30). **e.** Test predictions for an independent dataset from healthy donors older than 70 years (Old) and younger than 30 years (Young) using publicly available scRNA-seq datasets of $CD8^+$ T cells. The box-whisker plots in **d** and **e**, the center line is the median, the box is from the 25th to the 75th percentile. The upper or lower whisker extends from the hinge to the 1.5 * IQR from the hinge (where IQR is distance between the first and third quartiles) for up and low, respectively. P values were calculated by 2-sided T-test. **f**. Ridge plots showing the distribution of residuals from both mixed effect models used in the study.

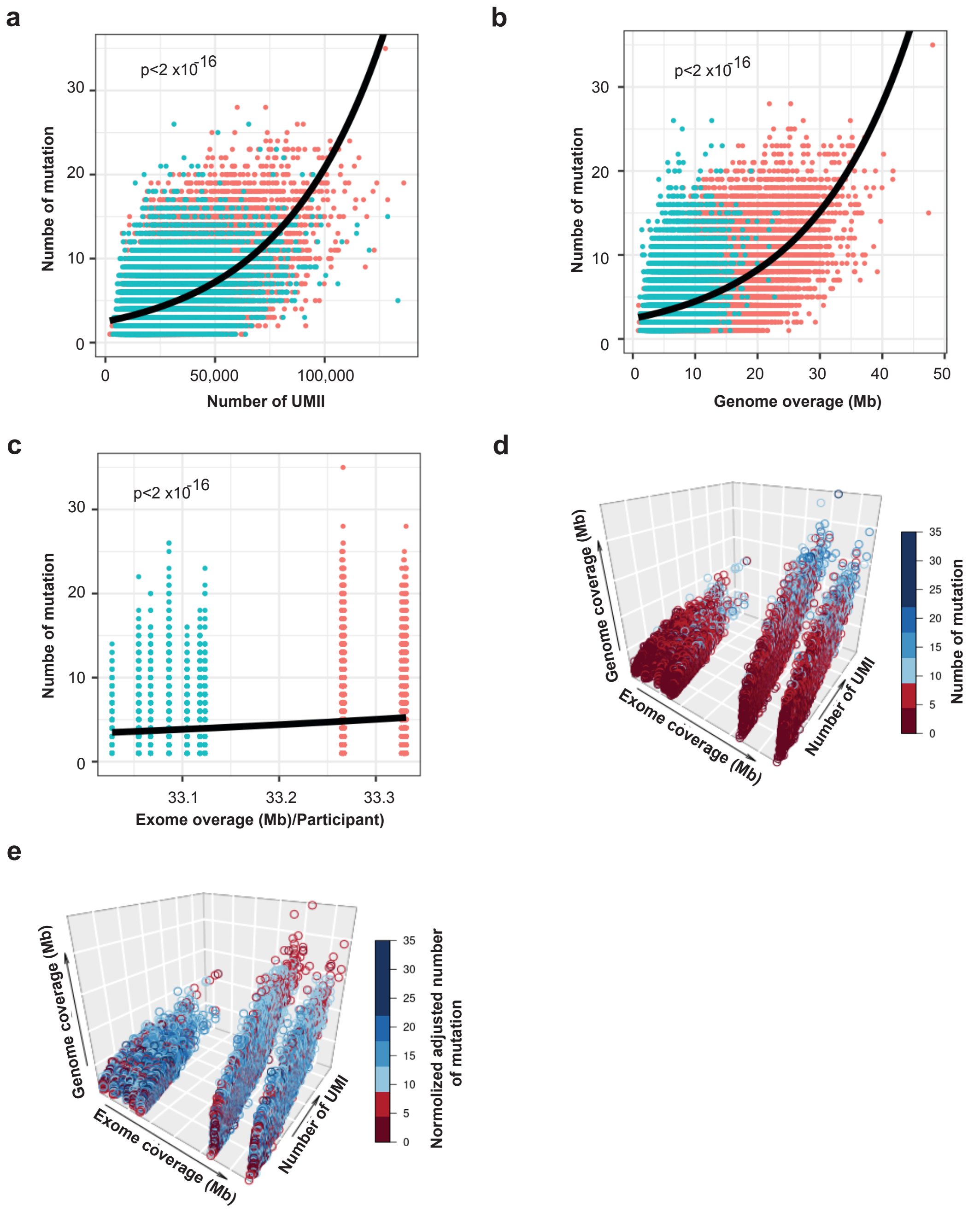

Supplementary Figure 6: Mutational analysis of $CD8^{+}$ T cell subpopulations and their change with age. **a.** Scatter plot showing the relationship between umi number and number of raw mutations in single cells. Fitted negative binomial regression line shown. Red color represents cross-sectional and blue represents longitudinal samples. **b.** Scatter plot showing the relationship between coverage and number of raw mutations in single cells. Fitted negative binomial regression line shown. **c.** Scatter plot showing the relationship between 10X coverage for exome sequencing for each donor and number of raw mutations in single cells. Fitted negative binomial regression line shown. **d**. 3D scatter plot showing the relationship between three variables and number of mutations in single cells. **e**. 3D scatter plot showing the relationship between three variables and number of scaled mutations in single cells after adjustment using the residuals from negative binomial regression.

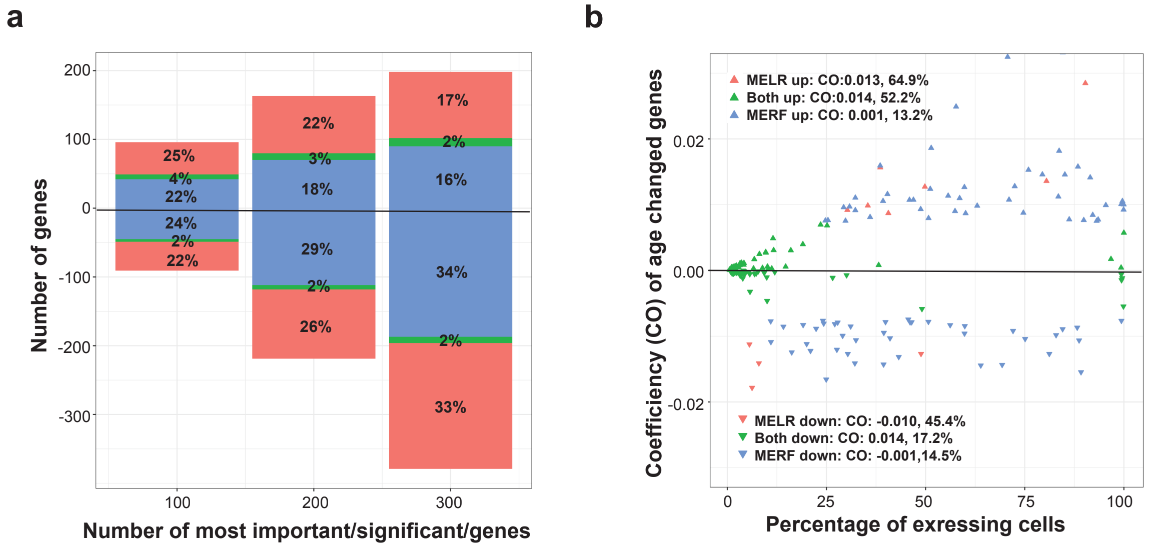

Supplementary Figure 7: a**.** Overlap of age-associated genes in CD8$^+$ T cells between MELR and MERF models. The percentages of overlaps were incremented by the top 100, 200, and 300 genes identified by MELR based on FDR and coefficient of age. Percentages are grouped by if the genes were up or downregulated across aging as identified by the MELR method. b**.** Scatter plot of the coefficient of age (CO) of age-associated genes identified by MELR and the percentages of cells expressing each gene. The average change (CO) and percentage of each six groups are presented.

Supplementary Table 1 Information of participants' age, gender, number of CD8 T cells, composition of CD8 T cell subpopulations by scRNAseq and coverage by exome sequencing

Donor ID	Age	Sex	CD8 T cell[a]	Cluster Percentages										Reads (M)[b]		Coverage (Mb)[c]	
				Nc	Na	SCM	CM	EM1	EM2	EM3	EMRA1	EMRA2	EFF	Exome	Genome	Exome	Genome
sc_d1	0	M	5185	0.96	0.00	0.00	0.00	0.00	0.00	0.00	0.00	0.00	0.04	109,496	151,321	33	117
sc_d2	0	F	3836	0.95	0.00	0.00	0.00	0.00	0.00	0.00	0.00	0.00	0.05	107,497	148,993	33	118
sc_d3	26	M	5525	0.00	0.64	0.02	0.05	0.06	0.01	0.15	0.04	0.03	0.01	115,366	156,665	33	116
sc_d4	28	F	4704	0.00	0.25	0.02	0.12	0.22	0.01	0.05	0.27	0.04	0.01	111,847	154,610	33	117
sc_d5	37	F	5591	0.00	0.32	0.03	0.17	0.12	0.01	0.05	0.29	0.01	0.01	104,399	142,859	33	115
sc_d6	46	M	6635	0.00	0.27	0.02	0.09	0.13	0.04	0.03	0.09	0.25	0.09	117,520	160,829	33	117
sc_d7	53	F	4351	0.00	0.39	0.04	0.12	0.36	0.01	0.01	0.04	0.02	0.01	108,834	150,416	33	117
sc_d8	64	M	4944	0.00	0.08	0.29	0.13	0.11	0.02	0.16	0.16	0.03	0.03	97,985	134,322	33	114
sc_d9	68	F	4323	0.00	0.32	0.06	0.05	0.07	0.01	0.45	0.00	0.00	0.03	112,257	153,600	33	117
sc_d10	76	F	5322	0.00	0.04	0.36	0.07	0.19	0.02	0.03	0.08	0.20	0.01	109,832	150,430	33	117
sc_d11	77	M	4899	0.00	0.31	0.10	0.13	0.14	0.02	0.06	0.10	0.11	0.02	114,630	156,209	33	116
sc_d12	83	M	4908	0.00	0.03	0.16	0.14	0.11	0.02	0.32	0.12	0.06	0.04	104,323	143,523	33	114
sc_d13	83	F	5839	0.00	0.04	0.12	0.23	0.29	0.02	0.03	0.08	0.11	0.09	103,256	141,517	33	113
sc_d14	84	M	4956	0.00	0.06	0.03	0.05	0.18	0.01	0.05	0.54	0.07	0.01	103,805	142,393	33	114
sc_d15	88	M	5217	0.00	0.04	0.09	0.11	0.30	0.02	0.07	0.09	0.27	0.01	116,460	159,003	33	117
sc_d16	90	F	6865	0.00	0.04	0.12	0.17	0.08	0.02	0.02	0.51	0.01	0.02	107,770	146,975	33	115
L1-1	30	F	2329	0.00	0.33	0.06	0.06	0.11	0.01	0.08	0.20	0.15	0.00	92,495	138,104	33	122
L1-2	39	F	1832	0.00	0.63	0.04	0.02	0.07	0.00	0.02	0.07	0.13	0.00				
L2-1	32	M	3878	0.00	0.25	0.14	0.07	0.19	0.04	0.07	0.10	0.14	0.00	120,847	175,192	33	132
L2-2	40	M	2030	0.00	0.23	0.13	0.08	0.24	0.03	0.09	0.13	0.07	0.00				
L3-1	48	M	2386	0.00	0.11	0.08	0.16	0.09	0.01	0.05	0.45	0.05	0.00	98,196	144,562	33	128
L3-2	56	M	3141	0.00	0.04	0.04	0.12	0.06	0.01	0.05	0.59	0.09	0.01				
L4-1	54	F	450	0.00	0.50	0.36	0.03	0.03	0.00	0.00	0.01	0.06	0.00	101,174	148,152	33	129
L4-2	65	F	5187	0.00	0.35	0.51	0.10	0.01	0.00	0.00	0.01	0.01	0.00				
L5-1	60	F	1414	0.00	0.09	0.08	0.10	0.19	0.03	0.08	0.17	0.25	0.01	102,113	170,473	33	143
L5-2	65	F	1536	0.00	0.12	0.04	0.03	0.04	0.01	0.04	0.06	0.66	0.01				
L5-3	70	F	2017	0.00	0.06	0.04	0.04	0.17	0.02	0.05	0.39	0.23	0.01				
L6-1	60	M	2217	0.00	0.06	0.07	0.11	0.12	0.02	0.05	0.34	0.23	0.00	102,005	151,478	33	128
L6-2	71	M	2091	0.00	0.08	0.04	0.05	0.07	0.01	0.01	0.38	0.35	0.00				
L7-1	69	F	118	0.00	0.03	0.04	0.01	0.03	0.03	0.40	0.11	0.26	0.08	91,381	135,938	33	130
L7-2	78	F	2632	0.00	0.08	0.18	0.04	0.26	0.12	0.11	0.06	0.14	0.00				
L8-1	69	M	2108	0.00	0.40	0.19	0.11	0.17	0.02	0.04	0.03	0.04	0.00	104,046	154,062	33	122
L8-2	77	M	1952	0.00	0.25	0.48	0.18	0.04	0.01	0.02	0.01	0.02	0.00				

[a] Cell number was determined by scRNAseq data

[b] Number of sequencing reads in each donor exome sequencing file

[c] Calculated as the number of basepairs that were covered by at least 10 sequencing reads (10X depth)

Supplementary Table 2 Selected enriched gene expression function in CD8 T cell subpopulations

Description	ID	setSize	NES	qvalues	Enriched genes (selected)	Number of enriched gene	CD8 T sobpopulation
RNA localization	GO:0006403	203	1.71	0.010	TGFBR2/CCT4/CCT3/TPR/CCT5/CCT6A/CCT2/UPF3A/RBM26/ATM/NPM1/NOP10/ZFP36L1	15	Nc
protein-DNA complex subunit organization	GO:0071824	165	1.55	0.025	PTMA/PARP1/NAP1L1/CAND1/MYC/NPM1/HMGA1/ANP32B/SET/RBX1/NAP1L4/RPL23/UBTF	13	Nc
telomere maintenance	GO:0000723	123	1.61	0.019	CCT4/CCT3/PARP1/CCT5/CCT6A/APEX1/CCT2/MYC/ATM/POLD4/HSP90AB1/NOP10	12	Nc
regulation of DNA biosynthetic process	GO:2000278	76	1.64	0.026	CCT4/CCT3/CCT5/CCT6A/RGCC/CCT2/MYC/ATM/HSP90AB1/ARRB2	10	Nc
methylation	GO:0032259	261	1.49	0.020	SATB1/MTERF4/DPY30/PARP1/PRMT2/MAT2B/MYC/DPH5/PRMT1/GSTO1	10	Nc
DNA conformation change	GO:0071103	183	1.61	0.010	TPR/PARP1/NAP1L1/SMC4/NPM1/HMGA1/ANP32B/SET/RBX1/NAP1L4	10	Nc
chromatin remodeling	GO:0006338	126	1.57	0.032	SATB1/PTMA/TPR/CBX3/MYC/NPM1/HMGA1/HDAC1	8	Nc
DNA packaging	GO:0006323	91	1.82	0.010	TPR/NAP1L1/SMC4/NPM1/HMGA1/ANP32B/SET/NAP1L4	8	Nc
cellular amino acid metabolic process	GO:0006520	200	1.46	0.035	PSMB4/PSMA5/SERINC5/PPM1K/EGLN2/PPA1/PSMA4	7	Nc
telomerase RNA localization to Cajal body	GO:0090671	19	1.92	0.010	CCT4/CCT3/CCT5/CCT6A/CCT2/NOP10	6	Nc
ribosome localization	GO:0033750	13	1.96	0.011	NPM1/RPS15	2	Na
chaperone-mediated autophagy	GO:0061684	12	1.88	0.034	HSPA8	1	SCM
negative regulation of proteasomal ubiquitin-dependent protein catabolic process	GO:0032435	29	1.92	0.018	RPL11	1	SCM
regulation of intrinsic apoptotic signaling pathw ay	GO:2001242	117	1.53	0.044	SOD1/TPT1/RPL11/MIF/CYLD	5	SCM
response to antibiotic	GO:0046677	168	1.49	0.049	TXNIP/SOD1/TNFAIP3/CDKN1B/KLF2/GATA3/ANXA1/LDHA/CRIP1/EEF2	10	SCM
export across plasma membrane	GO:0140115	12	1.95	0.020	FXYD2	1	CM
negative regulation of endothelial cell apoptotic process	GO:2000352	10	2.00	0.022	TNFAIP3	1	CM
T cell apoptotic process	GO:0070231	32	1.76	0.045	IL7R/TSC22D3	2	CM
homeostasis of number of cells	GO:0048872	164	1.57	0.020	IL7R/TNFAIP3/LYAR/FLT3LG/TSC22D3/BCL2	6	CM
negative regulation of transmembrane transport	GO:0034763	50	1.75	0.015	ARL6IP5/YWHAQ/BIN1/CALM2/SRI/TGFB1/CALM3/PRKCB/CALM1/OAZ1/SLC9A3R1	11	EM2
positive regulation of calcium-mediated signaling	GO:0050850	22	1.78	0.011	CALM2/CALM3/CIB1/CCL4/CALM1	5	EM2
regulation of lymphocyte apoptotic process	GO:0070228	34	1.75	0.020	CD74/CD27/CD3G/PRELID1/BAX/CCL5	6	EM2
regulation of phosphatase activity	GO:0010921	109	1.53	0.033	GNAI2/PPP1R7/PLEK/CALM2/PTPRC/ENSA/FKBP1A/PPP1R12A/WNK1/CALM3/CALM1	12	EM2
transcription elongation from RNA polymerase II promoter	GO:0006368	77	1.61	0.032	NELFCD/CCNH/POLR2J/ELOC/POLR2F/ELOB/POLR2G/RTF1/EAPP	9	EM2
protein polyubiquitination	GO:0000209	245	1.56	0.004	PSMA5/PSMD4/PSMB4/PSMA7/RBCK1/PSMB1/PSMA2/TNFAIP3/UBC/UBE2N/RNF19A	30	EM2
positive regulation of T cell proliferation	GO:0042102	53	1.68	0.023	PTPRC/HMGB1/HLA-DPA1/HLA-DPB1/HLA-E/ANXA1/CORO1A/PYCARD/CD81/CCL5	10	EM2
regulation of mRNA splicing, via spliceosome	GO:0048024	75	1.59	0.038	SRSF7/RBM8A/SON/SRSF9/RBM25/DDX5	6	EM2
regulation of nuclear division	GO:0051783	101	1.56	0.032	TPR/CHMP2A/TGFB1/CALR/PIN1/FBXW5/BUB3	7	EM2
G2/M transition of mitotic cell cycle	GO:0000086	183	1.73	0.003	TUBA4A/PSMA5/PSMD4/ENSA/PSMB4/PSMA7/CDC25B/CCNH/PSMB1/PSMA2	31	EM2
maintenance of protein localization in organelle	GO:0072595	34	1.73	0.027	SYNE1/PARK7/TXN/HSPA5/SUN2	5	EMRA2
phosphatidylcholine metabolic process	GO:0046470	38	1.65	0.049	DBI/CAPN2/PLAAT4	3	EMRA2
positive regulation of protein autophosphorylation	GO:0031954	13	1.73	0.038	IQGAP1/CALM1	2	EMRA2
positive regulation of protein complex disassembly	GO:0043243	23	1.70	0.046	PLEK/DSTN/WDR1/CFL1	4	EMRA2
regulation of focal adhesion assembly	GO:0051893	33	1.73	0.031	RHOA/ITGB1BP1/RAC1/MACF1/IQGAP1/ROCK1	6	EMRA2
covalent chromatin modification	GO:0016569	357	1.52	0.007	MECP2/ARID4B/ASH1L/USP16/KMT2E/USP15/ATM/RSF1/KMT2A/SFPQ/EPC1/ATRX	15	EFF
glycoprotein metabolic process	GO:0009100	233	1.60	0.007	RPN2/CHST12/ITM2B/TMEM165/TMEM59/SPOCK2/ITM2A/OGA/CCR7	9	EFF
mRNA export from nucleus	GO:0006406	102	1.60	0.031	SRSF7/TPR/HNRNPA2B1/DDX39B/SRSF11/UPF2/SRSF5/SRSF2	8	EFF
alpha-beta T cell proliferation	GO:0046633	22	2.16	0.007	ZAP70/PTPRC/IRF1/CD3E/HLA-E/RASAL3/VSIR	7	EFF
positive regulation of cytokine biosynthetic process	GO:0042108	33	1.81	0.016	PTPRC/CD74/IRF1/CD3E/SPN/STAT3	6	EFF
STAT cascade	GO:0097696	70	1.66	0.026	STAT4/PTPRC/IL7R/IL10RA/STAT3/PTPN2	6	EFF
negative regulation of lymphocyte mediated immunity	GO:0002707	21	2.13	0.007	PTPRC/IL7R/CD96/HLA-E/HLA-F	5	EFF
response to interleukin-7	GO:0098760	22	1.79	0.025	IL7R/IL2RG/STAT3/PDIA3/P4HB	5	EFF
histone H3-K9 modification	GO:0061647	32	1.78	0.028	MECP2/ARID4B/ASH1L/KMT2A/ATRX	5	EFF
T-helper cell differentiation	GO:0042093	40	1.74	0.030	FOXP1/ANXA1/SPN/STAT3/RORA	5	EFF

Supplementary Table 3. Antibodies used in this study

Antibodies	fluorochrome	Clone	Dilution	Company	Catalog#	Used in
CD8	FITC	HIT8a	1:20	BioLegend	300906	Longitudinal cell labeling and sorting
CD14	Pacific Blue	HCD14	1:20	BioLegend	325616	
TotalSeq™-A0386 CD28	N/A	CD28.2	1:100	BioLegend	302955	
TotalSeq™-A0063CD45RA	N/A	HI100	1:100	BioLegend	304157	
CD3	BV510	OKT3	1:20	BioLegend	317332	Cross sectional Cell sorting
CD19	BV421	HIB19	1:20	BioLegend	302234	
CD4	PE	OKT4	1:20	BioLegend	317410	
CD8	FITC	HIT8a	1:20	BioLegend	300906	
B2M	BB515	2M2	1:100	BioLegend	316304	Multi-color phenotyping
CD127	BV711	A019D5	1:50	BioLegend	351328	
CD18	AF700	TS1/18	1:100	BioLegend	302124	
CD184	BUV737	12G5	1:1000	BD	741862	
CD2	BV605	RPA-2.10	1:100	BioLegend	300224	
CD27	BUV395	L128	1:20	BD	563815	
CD28	BV785	CD28.2	1:50	BioLegend	302950	
CD28	BV711	CD28.2	1:100	BD	563131	
CD3	BV570	UCHT1	1:50	BioLegend	300436	
CD4	BB700	SK3	1:1000	BD	566393	
CD45RA	PE-Cy7	HI100	1:100	BioLegend	304126	
CD45RO	BV605	UCHL1	1:100	BD	562791	
CD62L	APC	DREG-56	1:100	BioLegend	304809	
CD62L	BV786	DREG-56	1:100	BioLegend	304830	
CD69	BV650	FN50	1:20	BioLegend	310934	
CD8	BUV496	RPA-T8	1:50	BD	564804	
CD95	PE/Cy5	DX2	1:100	BioLegend	305610	
EOMES	eF710	WD1928	1:100	eBioscience	44-4877-42	
GZMA	APC	CB9	1:20	BioLegend	507220	
GZMB	AF700	GB11	1:100	BD	560213	
KLRG1	PE	2F1/KLRG1	1:20	BioLegend	138408	
PD-1	BUV737	EH12.1	1:20	BD	565299	
Perforin	PE-CF594	8G9	1:100	BD	563763	

Supplementary Table 4 Sequences of primers used to construct antibody-derived tag (ADT) libraries

Primer name	Primer sequences	Source
R2-PCR handle primer for 1st round	5’-GTGACTGGAGTTCAGACGTGTCCTTGGCACCCGAGAATTCC-3’	IDT
Reverse primer for 1st round	5’-AATGATACGGCGACCACCGA-GATCTACACTCTTTCCCTACACGACGCTC-3’	IDT
P5 forward primer for 2nd round	5’-AATGATACGGCGACCACCGAGA-TCTACACTCTTTCCCTACACGACGCTC-3’	IDT
Individual sample index primer for 2nd round	5’-CAAGCAGAAGACGGCATACGAGAT-NNNNNNNN-GTGACTGGAGTTCAGACGTGT-3‘	10xGenomics
