# Supplementary material for: Heterogeneity and transcriptome changes of human CD8+ T cells across nine decades of life
Source: Nat Commun. 2022 Sep 1;13:5128. doi: 10.1038/s41467-022-32869-x (PMC9436929; doi:10.1038/s41467-022-32869-x)
Supplement: Supplementary file 3 — Reporting Summary [file 41467_2022_32869_MOESM3_ESM.pdf]

nature portfolio

Corresponding author(s): Nan-ping Weng

Last updated by author(s): 2022-5-14

Reporting Summary

Nature Portfolio wishes to improve the reproducibility of the work that we publish. This form provides structure for consistency and transparency in reporting. For further information on Nature Portfolio policies, see our Editorial Policies and the Editorial Policy Checklist.

Statistics

For all statistical analyses, confirm that the following items are present in the figure legend, table legend, main text, or Methods section.

n/a	Confirmed	
☐	☒	The exact sample size (*n*) for each experimental group/condition, given as a discrete number and unit of measurement
☐	☒	A statement on whether measurements were taken from distinct samples or whether the same sample was measured repeatedly
☐	☒	The statistical test(s) used AND whether they are one- or two-sided<br>*Only common tests should be described solely by name; describe more complex techniques in the Methods section.*
☐	☒	A description of all covariates tested
☐	☒	A description of any assumptions or corrections, such as tests of normality and adjustment for multiple comparisons
☐	☒	A full description of the statistical parameters including central tendency (e.g. means) or other basic estimates (e.g. regression coefficient) AND variation (e.g. standard deviation) or associated estimates of uncertainty (e.g. confidence intervals)
☐	☒	For null hypothesis testing, the test statistic (e.g. *F*, *t*, *r*) with confidence intervals, effect sizes, degrees of freedom and *P* value noted<br>*Give P values as exact values whenever suitable.*
☒	☐	For Bayesian analysis, information on the choice of priors and Markov chain Monte Carlo settings
☒	☐	For hierarchical and complex designs, identification of the appropriate level for tests and full reporting of outcomes
☐	☒	Estimates of effect sizes (e.g. Cohen's *d*, Pearson's *r*), indicating how they were calculated

Our web collection on statistics for biologists contains articles on many of the points above.

Software and code

Policy information about availability of computer code

Data collection	The scRNAseq data generated for this study are available with accession code GSE136184. Additional datasets were downloaded from public repositories. Data to validate our age prediction model were downloaded from the European Genome-Phenome Archive (accession EGAD00001006000) and from the 10X Genomics datasets website (https://www.10xgenomics.com/resources/datasets) that used 3' chemistry and included age information. For prediction on clinical datasets, data was downloaded from the Gene Expression Omnibus (accession codes 123412341234 and 123412341234).
Data analysis	10X Genomics Cell Ranger v4.0, R v3.6.2, Seurat v3.0.0, FlowJo v10.3, lme4 v1.1-27.1, glmnet v4.1-3, ranger v0.13.1, the script files to perform all analysis to reproduce the data/results in the paper as well as recreate all figures are deposited at GitHub (https://zenodo.org/badge/ latestdoi/364361401), and the somatic mutation identification pipeline (USCMD) is deposited at https://zenodo.org/badge/latestdoi/390409912.

For manuscripts utilizing custom algorithms or software that are central to the research but not yet described in published literature, software must be made available to editors and reviewers. We strongly encourage code deposition in a community repository (e.g. GitHub). See the Nature Portfolio guidelines for submitting code & software for further information.

Data

Policy information about availability of data

All manuscripts must include a data availability statement. This statement should provide the following information, where applicable:

- Accession codes, unique identifiers, or web links for publicly available datasets
- A description of any restrictions on data availability
- For clinical datasets or third party data, please ensure that the statement adheres to our policy

The scRNAseq data generated for this study are available at GSE136184.

Field-specific reporting

Please select the one below that is the best fit for your research. If you are not sure, read the appropriate sections before making your selection.

☒ Life sciences ☐ Behavioural & social sciences ☐ Ecological, evolutionary & environmental sciences

For a reference copy of the document with all sections, see nature.com/documents/nr-reporting-summary-flat.pdf

Life sciences study design

All studies must disclose on these points even when the disclosure is negative.

Sample size

24 human donors (33 samples) were recruited for this study. This number was chosen to include donors that covered the spectrum of human aging in each decade of life from 0 to 90 years, including both sexes in each decade of life. Due to the difficulty in collecting human samples, we believe this number is a significant amount of donor samples for this study.

Data exclusions

No data were excluded from the analysis.

Replication

The method of scRNAseq was reproducible for same samples.

Randomization

The donors were chosen based on ages and sexes that fell into specific decades of life to cover the spectrum of human aging and have equal amounts of male and female donors. Variables that were controlled for in statistical analyses included sex.

Blinding

Investigators were not blinded to the age and sex of the study subjects. However, investigators were blinded to all other patient or sample characteristics. Sex was controlled for using various statistical methods in each analysis.

Reporting for specific materials, systems and methods

We require information from authors about some types of materials, experimental systems and methods used in many studies. Here, indicate whether each material, system or method listed is relevant to your study. If you are not sure if a list item applies to your research, read the appropriate section before selecting a response.

Materials & experimental systems

n/a	Involved in the study	
☐	☒	Antibodies
☒	☐	Eukaryotic cell lines
☒	☐	Palaeontology and archaeology
☒	☐	Animals and other organisms
☐	☒	Human research participants
☒	☐	Clinical data
☒	☐	Dual use research of concern

Methods

n/a	Involved in the study	
☒	☐	ChIP-seq
☐	☒	Flow cytometry
☒	☐	MRI-based neuroimaging

Antibodies

Antibodies used

All antibodies used are listed in the Supplemental Table S4

Validation

All antibodies were validated by company at their website.

Human research participants

Policy information about studies involving human research participants

Population characteristics

Human adult donors were selected based on ages that represented each decade of life from 20 to 90 years old. Cord blood was also collected from 2 donors. Participants in each decade were matched by sex (e.g. there were two donors - 1 female and 1 male - within the age range 20 to 30).

Recruitment

Participants were voluntary blood donors. It has been reported there is a "healthy donor effect" where healthier donors are more likely to donate blood. However, this study enlisted healthy donors, and therefore, this effect should not affect the results.

Ethics oversight

All studies were approved by the National Institutes of Health Institutional Review Board and the Johns Hopkins Bayview Institutional Review Board.

Note that full information on the approval of the study protocol must also be provided in the manuscript.

Flow Cytometry

Plots

Confirm that:

- [X] The axis labels state the marker and fluorochrome used (e.g. CD4-FITC).
- [X] The axis scales are clearly visible. Include numbers along axes only for bottom left plot of group (a 'group' is an analysis of identical markers).
- [X] All plots are contour plots with outliers or pseudocolor plots.
- [X] A numerical value for number of cells or percentage (with statistics) is provided.

Methodology

Sample preparation	Peripheral blood mononuclear cells (PBMCs) were isolated from human blood by Ficoll density gradient centrifugation. For adult donors in the cross-sectional cohort, CD8+ cells were enriched from PBMCs using an EasySep Human CD8 Positive Selection Kit II (STEMCELL Technologies) according to the manufacturer's instructions. Cells were resuspended in phosphate buffered saline (PBS) containing 0.04% bovine serum albumin (BSA). Purity was over 95% for all samples. PBMCs from cord blood were cryopreserved and thawed on the day of use in warm media for 1 hour, washed once with BSM (HBSS containing 0.2% BSA, 1X HEPES, 1X penicillin-streptomycin-glutamine), and stained with fluorescent antibodies against CD3, CD4, and CD8. CD8+ cells were sorted as CD8+/CD4- cells into PBS containing 0.04% BSA. For longitudinal cohort samples, cryopreserved PBMCs were thawed on the day of use in warm media for 1 hour, washed once with BSM, and stained with antibody cocktails containing fluorescent antibodies against CD8 and CD4, and DNA barcoded antibodies against CD28 and CD45RA (Supplementary Fig 1c). CD8+ cells were sorted as CD8+/CD4- cells into PBS containing 0.04% BSA.
Instrument	BD FACSymphony flow cytometer (BD Biosciences)
Software	The flow cytometry data was analyzed using FloJo v10.3.
Cell population abundance	CD8+ cells were sorted as CD8+ cells and had purities ranging from 97% to 98% in collected fractions.
Gating strategy	Cells were gated as standard by viability gate, single cell gate, and CD8+ gate. All CD8+ cells were analyzed by FlowSOM.

- [X] Tick this box to confirm that a figure exemplifying the gating strategy is provided in the Supplementary Information.
